# Phase I Pilot Study of a Novel Celecoxib-Containing Paste for Local Application Following Mandibular Third Molar Extraction: Safety, Tolerability, and Feasibility of a Dose-Escalation Design

**DOI:** 10.7759/cureus.112213

**Published:** 2026-07-07

**Authors:** Yasumasa Kakei, Masaya Akashi, Takumi Hasegawa, Takeshi Ioroi, Ikuko Yano

**Affiliations:** 1 Oral and Maxillofacial Surgery, Kobe University Hospital, Kobe, JPN; 2 Pharmacy, Kobe University Hospital, Kobe, JPN

**Keywords:** celecoxib, cox-2 inhibitor, dose escalation, local drug delivery, mandibular third molar extraction, phase i study, postoperative pain, third molar extraction

## Abstract

Background and objectives: Postoperative pain after mandibular third molar extraction remains a significant clinical challenge. Local drug delivery to the extraction socket may enable targeted analgesia with minimal systemic exposure. This first-in-human pilot study aimed to evaluate the safety and tolerability of a novel celecoxib-containing paste applied to extraction sockets and to assess the feasibility of the dose-escalation design, outcome measures, and study procedures to inform a future definitive randomized controlled trial.

Methodology: This single-center, open-label, dose-escalation study enrolled 18 adult patients undergoing mandibular third molar extraction in three sequential cohorts (n = 6 each): low (0.5 mg/mL), medium (1.0 mg/mL), and high (2.0 mg/mL) celecoxib paste. Following extraction, 1-2 mL of paste was applied to the socket. The primary safety outcome was the incidence of alveolar osteitis (dry socket) by day 8. Feasibility outcomes included recruitment and retention rates, protocol adherence, and the performance of exploratory efficacy measures, including the visual analog scale (VAS) pain scores, rescue analgesic consumption, and patient-reported effectiveness. The study followed the Consolidated Standards of Reporting Trials (CONSORT) extension for pilot and feasibility trials, adapted for a non-randomized design.

Results: All 18 participants completed the study with no withdrawals (retention rate: 100%). No cases of dry socket or treatment-related adverse events were observed (0/18; 95% CI: 0-15.3%). Mean VAS pain scores declined consistently across all cohorts from days 1 to 5. Higher-dose groups showed numerically greater reductions in rescue analgesic consumption. The dose-escalation protocol, outcome measures, and data collection procedures were feasible and performed as intended.

Conclusions: Application of celecoxib-containing paste to extraction sockets was well tolerated without dry socket or other safety concerns. The study procedures were feasible, and preliminary efficacy signals support progression to a larger, randomized, placebo-controlled trial.

Trial registration: Japan Registry of Clinical Trials (jRCT; https://jrct.niph.go.jp/; identifier: jRCT2051230162). Registered on January 11, 2024.

## Introduction

Third molar extraction is a frequent oral surgical procedure [1,2]. It can be highly invasive during bone removal or crown sectioning, which results in moderate to severe postoperative complications, including pain, edema, trismus, and dysphagia [2,3] that may significantly impair oral intake following surgery [4].

Post-extraction pain is a prevalent and clinically important postoperative complication, which is often the primary reason for avoiding treatment among patients [1]. Non-steroidal anti-inflammatory drugs (NSAIDs), acetaminophen, and traditional herbal medicines (such as Rikkosan in Japan) are approved for post-extraction pain management. Current regimens include celecoxib (200 mg) [4-6], ibuprofen (200-600 mg) [6,7], acetaminophen (500-1000 mg), opioid combinations (e.g., tramadol/acetaminophen) [8], and ibuprofen/acetaminophen combinations [9]. However, despite these available options, postoperative pain control remains suboptimal in many patients, highlighting an ongoing need for improved analgesic strategies.

When prescribing postoperative analgesics, it is essential to carefully consider potential adverse effects (AEs). Opioid-related concerns include respiratory depression, nausea, vomiting, and constipation [10], while NSAIDs may cause gastrointestinal disorders, renal and hepatic dysfunction, and platelet aggregation inhibition [11]. These systemic side effects underscore the clinical rationale for exploring locally delivered formulations that may achieve therapeutic tissue concentrations at the surgical site while reducing systemic exposure [12-14].

The extraction socket environment is characterized by the loss of keratinized mucosa and facilitates rapid local drug absorption at the affected site, which potentially enables targeted therapeutic effects. Our previous phase II, placebo-controlled, double-blind, randomized crossover study evaluated an ibuprofen gargle for post-extraction pain and demonstrated significant analgesia compared with placebo, providing proof-of-concept for local NSAID delivery to the socket [15].

Cyclooxygenase-2 (COX-2) inhibitors represent a class of NSAIDs that specifically target the enzyme primarily responsible for inflammation and pain mediation [16]. These agents reduce the gastrointestinal ulceration risk associated with non-selective NSAIDs and minimize upper gastrointestinal bleeding complications [17]. In this study, we developed a novel paste formulation containing celecoxib for sustained application directly to the extraction socket. The primary aim of this pilot study was to evaluate the safety of the paste across three dose levels, with particular attention to the occurrence of alveolar osteitis (dry socket). A secondary aim was to assess the feasibility of the dose-escalation design, recruitment procedures, outcome measures, and data collection processes to inform the design of a future definitive randomized controlled trial (RCT) [18].

## Materials and methods

Study design and setting

**Figure 1:**
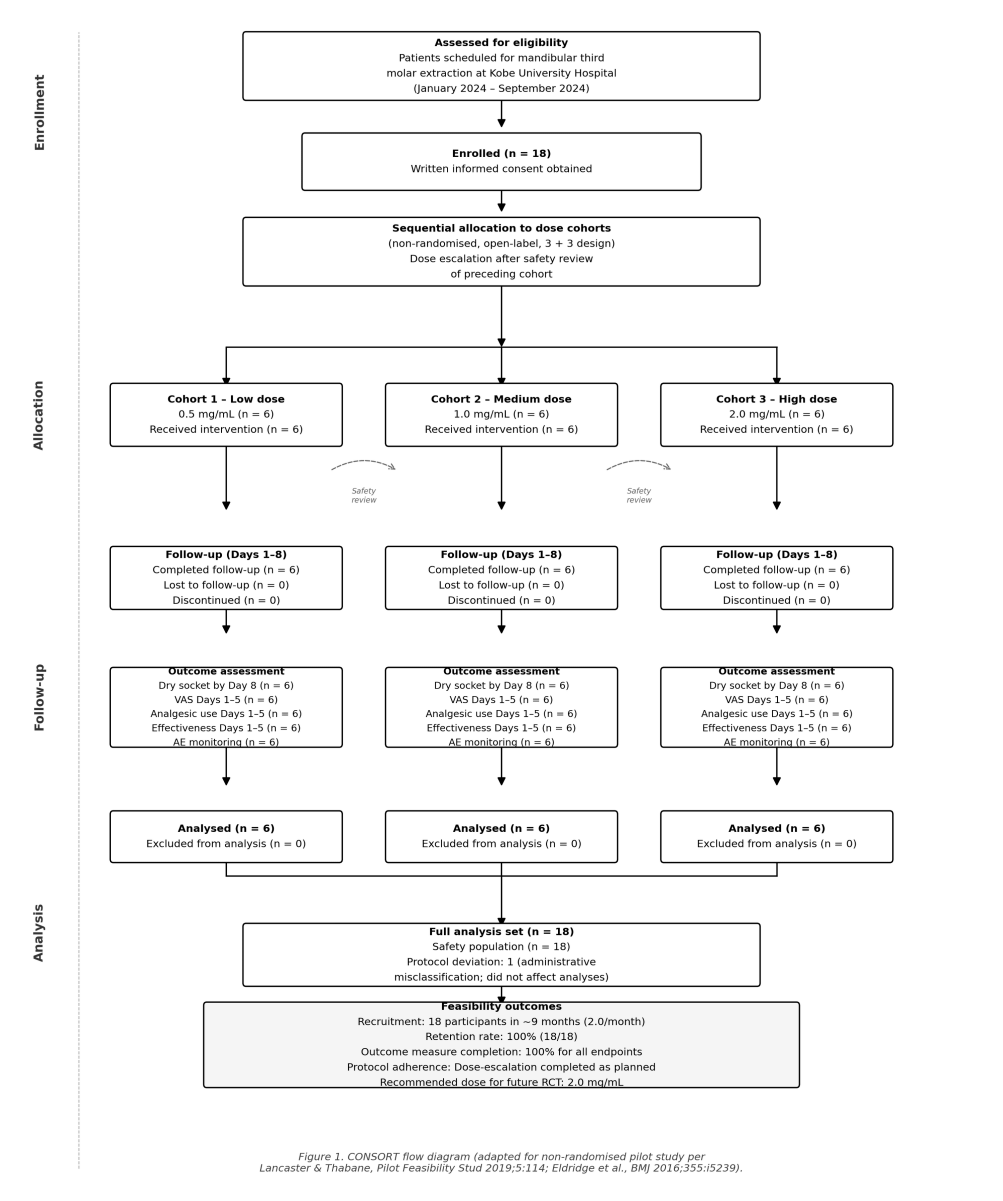
CONSORT flow diagram. Consolidated Standards of Reporting Trials (CONSORT) flow diagram adapted for a non-randomized pilot study [18]. DLT, dose-limiting toxicity; VAS: visual analog scale; AE: adverse effects; RCT: randomized controlled trial.

This was a non-randomized, prospective, open-label, single-center, dose-escalation pilot study for patients undergoing mandibular third molar extraction at Kobe University Hospital. The study commenced on January 11, 2024, and the completion date (final visit of the last patient) was September 30, 2024. The study is reported in accordance with the Consolidated Standards of Reporting Trials (CONSORT) extension for randomized pilot and feasibility trials [18], adapted for a non-randomized study design. Figure 1 presents the study protocol summary.

As this was a first-in-human dose-finding study intended to establish safety and feasibility rather than comparative efficacy, participants were allocated sequentially to pre-defined dose cohorts; accordingly, randomization, blinding, and allocation concealment were not applicable to this study design.

Objectives

The primary objective was to evaluate the safety of the celecoxib-containing paste across three dose levels, defined by the incidence of alveolar osteitis (dry socket) by day 8 post-extraction. Secondary objectives included: (1) assessment of feasibility parameters, including recruitment rate, retention, protocol adherence, and the performance of planned outcome measures; and (2) exploratory evaluation of analgesic efficacy (pain scores, rescue analgesic consumption, and patient-reported effectiveness) to inform endpoint selection and sample size estimation for a future definitive RCT.

Study population

Inclusion Criteria

Patients were eligible if they met all of the following criteria: (1) scheduled for mandibular third molar extraction with registration on the day of extraction; (2) teeth classified as Pell-Gregory class I or II (horizontal position) and position A or B (vertical position); (3) age ≥18 years at the time of registration; and (4) provision of written informed consent.

Exclusion Criteria

Patients were excluded if they met any of the following criteria: (1) severe or uncontrolled concomitant medical conditions; (2) history of hypersensitivity to selective COX-2 inhibitors, Tween 80, hydroxypropyl cellulose, or PEG400; (3) aspirin-exacerbated respiratory disease; (4) regular analgesic use for chronic pain; (5) current use of anticoagulants; (6) pregnancy, breastfeeding, or planning to become pregnant; and (7) other conditions deemed unsuitable by the investigator.

Intervention

Participants were enrolled sequentially into three dose cohorts following a traditional 3 + 3 design with predetermined safety stopping rules: cohort 1 (low dose), 0.5 mg/mL celecoxib paste (n = 6); cohort 2 (medium dose), 1.0 mg/mL celecoxib paste (n = 6); and cohort 3 (high dose), 2.0 mg/mL celecoxib paste (n = 6). Dose escalation proceeded only after confirmation of acceptable safety in the preceding cohort.

The celecoxib paste was formulated as a semi-solid preparation containing celecoxib as the active ingredient at specified concentrations, with excipients including hydroxypropyl cellulose, polyethylene glycol 400, and purified water; the pH was adjusted to 6.5-7.5 for optimal stability and tolerability. This novel formulation was developed to achieve sustained local drug release within the extraction socket. Prior to this first-in-human application, the formulation underwent pharmaceutical stability testing to confirm its physicochemical stability, and an in vivo safety study was performed in mice under the intended conditions of administration, in which no safety signals were identified. Moreover, celecoxib is an approved, commercially marketed drug, and the investigational paste was prepared by diluting it to concentrations well below systemic therapeutic levels; each applied dose contained less than 1/100 of the maximum approved daily oral dose of celecoxib, so that the anticipated safety risk was low.

All extractions were performed by experienced oral surgeons using standardized surgical techniques. Following local anesthesia with 2% lidocaine containing 1:80,000 epinephrine, a mucoperiosteal flap was raised, with bone removal and tooth sectioning performed as needed, and the tooth was extracted. Following wound closure, 1-2 mL of celecoxib paste at the assigned concentration was applied to the extraction socket using a disposable syringe with a blunt tip. Patients were instructed to retain the paste without rinsing or eating for at least 30 minutes.

Outcome measures

The primary outcome was the presence of alveolar osteitis (dry socket) by day 8 (%). Secondary outcomes included: (1) daily analgesic consumption (loxoprofen, 60 mg tablets) on postoperative days 1-5; (2) patient-reported overall effectiveness ratings (five-point scale: very good, good, fair, poor, very poor); (3) AEs and serious AEs (SAEs); and (4) daily overall pain assessed using a visual analog scale (VAS, 0-100 mm) on postoperative days 1-5. The data collection schedule is presented in Table 1.

**Table 1:** Data collection schedule. *1: The same day as day 1 is acceptable. *2: Postoperative data. *3: Sutures are removed on the same day.

Time - Item	Screening period	Registration	Treatment phase	Observation period					Time of discontinuance
			Day 1	Day 2	Day 3	Day 4	Day 5	Day8^*3^	
Allowance (Day)	−28 to 0	0	0	0	0	0	0	−2 to +4	0
Informed consent	●								
Patient demographics/medical history	●								
Registration		●^*1^							
Tooth extraction			●						
Test drug administration			●						
Test drug dosage			●						
Visual analog scale (VAS) value			●^*2^	●	●	●	●		
Painkillers: duration of use			●^*2^	●	●	●	●		
Painkillers: number of times used			●^*2^	●	●	●	●		
Overall effectiveness			●^*2^	●	●	●	●		
With or without dry socket								●	
Adverse events, etc.			●^*2^	●	●	●	●	●	●

Feasibility Outcomes

Pre-specified feasibility outcomes included: (1) recruitment rate (number of participants enrolled per month); (2) retention rate (proportion of enrolled participants completing all study visits through day 8); (3) protocol adherence (proportion of participants with complete data collection at all scheduled time points, and successful dose escalation according to stopping rules); and (4) acceptability of outcome measures (completion rates for VAS pain scores, analgesic diaries, and effectiveness ratings). These feasibility data were collected to inform the design parameters of a planned future definitive RCT.

Statistical analysis

A total of 18 patients were planned, with six patients per dose group. The sample size was determined based on a target toxicity level of 33%, adapted from phase I oncology trial methodology. The full analysis set served as the primary efficacy analysis population and the safety population. Continuous variables were summarized as mean ± standard deviation (SD), and categorical variables as frequencies and percentages. The primary outcome was analyzed using an exact 95% confidence interval (CI) for a binomial proportion. To visualize the trajectory of daily VAS pain scores while accounting for the repeated-measures structure of the data, a linear mixed-effects model was fitted with dose group, day (categorical), and their interaction as fixed effects, and a random intercept for each participant. Least-squares means (LS-means) and 95% CIs were estimated from this model using the means package and are presented.

The corresponding descriptive statistics (mean ± SD) are reported in the text. Dose-response relationships were evaluated descriptively across cohorts. As this was a pilot study, no formal hypothesis testing was performed; rather, results were intended to generate descriptive data and preliminary effect estimates to inform the design of a future definitive trial.

Ethics statement

The study complied with the principles of the Declaration of Helsinki (2013 revision), the Clinical Trials Act of Japan, and all applicable regulatory requirements. The study protocol was authorized by the Kobe University Hospital Certified Review Board (approval number: C230005) (https://jrct.mhlw.go.jp/latest-detail/jRCTs051230162). Written informed consent was obtained from all participants prior to enrollment. This was an investigator-initiated study conducted under the Clinical Trials Act of Japan, which was the applicable regulatory framework. Under this framework, review and approval of the protocol by a certified review board (CRB) is required, whereas separate approval by the national regulatory authority (the Pharmaceuticals and Medical Devices Agency, PMDA) is not required for this category of study. The trial was prospectively registered in the Japan Registry of Clinical Trials (jRCT2051230162).

## Results

Participant disposition and baseline characteristics

All 18 participants received treatment and were included in the analysis (n = 6 per cohort). Eighteen cases were enrolled between April 1, 2024, and August 14, 2024 (six in the low-dose group, six in the medium group, and six in the high-dose group). There were no withdrawals. Baseline demographic and clinical characteristics were well-balanced across dose cohorts (Table 2).

**Table 2:** Baseline characteristics by dose group.

Characteristic	Low dose (n = 6)	Medium dose (n = 6)	High dose (n = 6)	Total (n = 18)
Age, years, mean ± SD	26.2 ± 4.9	26.0 ± 4.7	26.5 ± 5.5	26.2 ± 4.7
Female, n (%)	2 (33.3)	2 (33.3)	4 (66.7)	8 (44.4)
Pell–Gregory classification (Class)				
Class I, n (%)	1 (16.7)	1 (16.7)	2 (33.3)	4 (22.2)
Class II, n (%)	5 (83.3)	5 (83.3)	4 (66.7)	14 (77.8)
Class III, n (%)	0 (0.0)	0 (0.0)	0 (0.0)	0 (0.0)
Pell–Gregory classification (Position)				
Position A, n (%)	5 (83.3)	2 (33.3)	4 (66.7)	11 (61.1)
Position B, n (%)	1 (16.7)	4 (66.7)	2 (33.3)	7 (38.9)
Position C, n (%)	0 (0.0)	0 (0.0)	0 (0.0)	0 (0.0)
Surgical time, min, mean ± SD	35.3 ± 9.3	41.3 ± 17.1	55.8 ± 19.6	44.2 ± 17.4

A protocol deviation occurred during cohort progression (administrative misclassification of one participant's dose cohort), which was documented and did not affect safety evaluations.

Feasibility outcomes

Recruitment was completed within approximately nine months. The retention rate was 100% (18/18), with all participants completing all scheduled visits through day 8. Protocol adherence was high: the dose-escalation procedure proceeded as planned, with successful safety review before each dose increment. Completion rates for outcome measures were as follows: VAS pain diaries 100% (18/18), rescue analgesic consumption diaries 100% (18/18), and patient-reported effectiveness ratings 100% (18/18). These results demonstrate that the study design, recruitment strategy, and outcome measures are feasible for a future definitive trial.

Primary Endpoint

The primary endpoint was dry socket through day 8. No dry socket cases occurred (0/18). The exact 95% CI for 0 events in 18 participants was 0-15.3%.

Secondary Outcomes

Safety: No AEs or SAEs were observed during follow-up.

**Figure 2:**
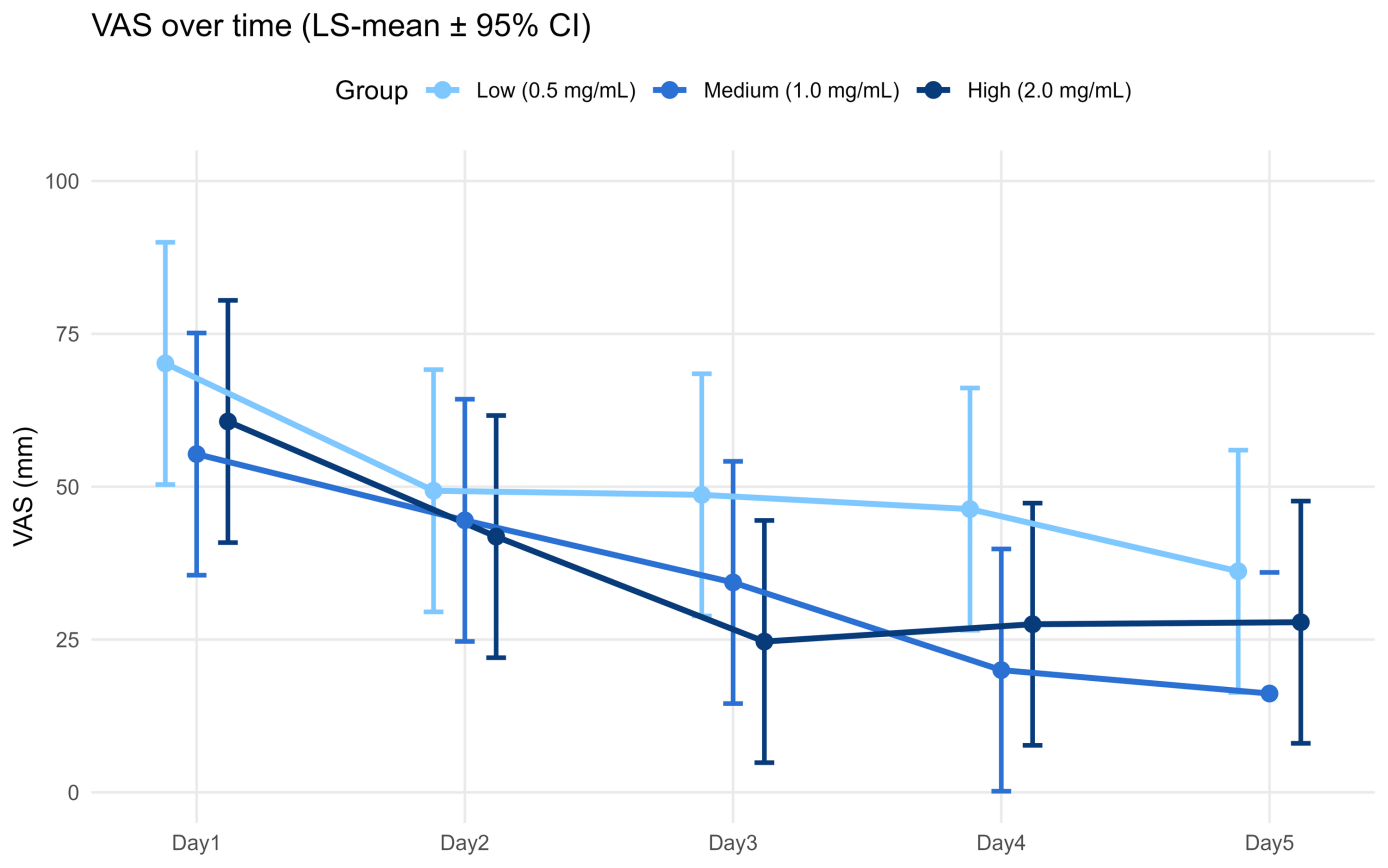
VAS pain scores over time (LS-mean ± 95% CI). LS, least-squares; CI, confidence interval; VAS, visual analog scale.

Daily overall pain (VAS, 0-100 mm; days 1-5): Mean VAS declined from day 1 to day 5 across cohorts (Figure 2): 0.5 mg/mL: 70.2 ± 15.0 → 36.2 ± 27.9 (Δ −34.0 mm); 1.0 mg/mL: 55.3 ± 30.6 → 16.2 ± 28.2 (Δ −39.2 mm); 2.0 mg/mL: 60.7 ± 12.2 → 27.8 ± 21.4 (Δ −32.8 mm).

**Figure 3:**
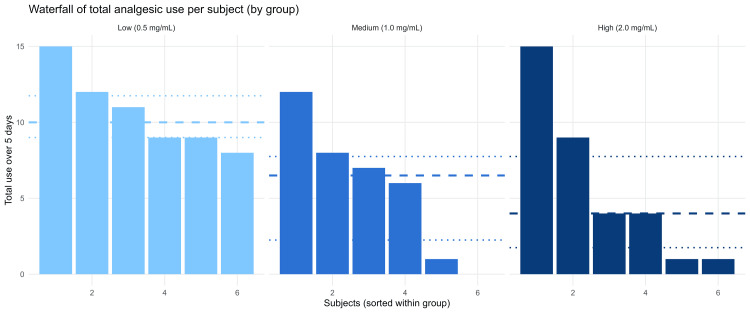
Waterfall plot of total analgesic use per subject (days 1-5). Within each treatment group (facet), subjects are ordered by decreasing total use. Bars show the cumulative number of as-needed analgesic intakes per subject. Horizontal dashed and dotted lines indicate the group median and interquartile range, respectively.

Rescue analgesic use (days 1-5): The total analgesic use per subject over postoperative days 1-5 is presented as a waterfall plot in Figure 3.

The median total number of uses was 10.0 (interquartile range (IQR): 9.0-11.8) in the low-dose group, 6.5 (IQR: 2.25-7.75) in the medium-dose group, and 4.0 (IQR: 1.75-7.75) in the high-dose group, suggesting a dose-dependent trend in rescue analgesic reduction.

**Figure 4:**
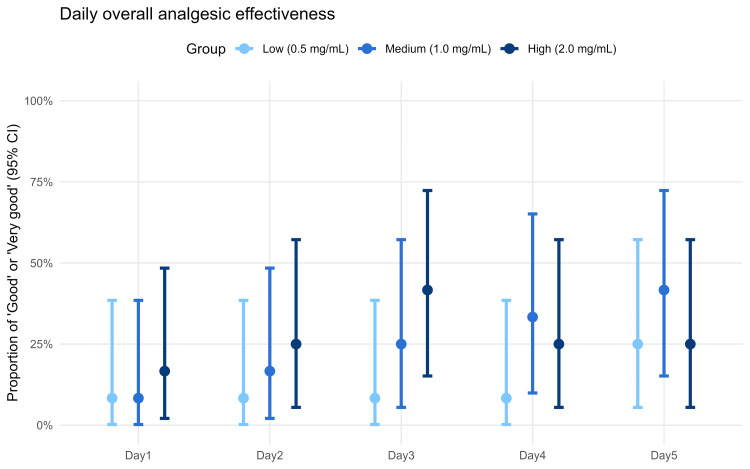
Daily overall analgesic effectiveness. Proportion of patients rating effectiveness as “good” or “very good” on each day. CI, confidence interval.

Patient-reported effectiveness: The overall daily effectiveness reported by patients (five-point Likert scale; days 1-5) and the proportion of patients rating effectiveness as “good” or “very good” increased over time in each cohort (Figure 4).

By day 5, these proportions reached 50.0% (95% CI: 11.8-88.2%) in the low-dose cohort and 83.3% (95% CI: 35.9-99.6%) in the medium- and high-dose cohorts.

## Discussion

This first-in-human, open-label, single-arm pilot study demonstrates that the application of a selective COX-2 inhibitor paste to mandibular third molar extraction sockets is well tolerated. There was no incidence of dry socket through day 8 in 18 participants (95% CI: 0-15.3%), with no treatment-related AEs across the three dose levels tested. Importantly, the study also demonstrated the feasibility of the dose-escalation design, recruitment procedures, and outcome measures, providing essential information for the planning of a definitive RCT.

From a feasibility perspective, several key findings emerged. First, the 100% retention rate and complete data collection across all outcome measures indicate that the study protocol is acceptable to patients and practical to implement. Second, the dose-escalation procedure using a 3 + 3 design with safety-stopping rules functioned as intended, demonstrating that this approach is appropriate for future dose-finding studies of intraoral formulations. Third, the exploratory efficacy measures (VAS pain scores, rescue analgesic diaries, and patient-reported effectiveness ratings) all showed consistent, interpretable patterns across dose groups, confirming their suitability as endpoints for a definitive trial.

Among the three dose levels, the exploratory efficacy signals were most favorable in the high-dose (2.0 mg/mL) cohort, which showed the greatest reduction in rescue analgesic consumption (median 4.0 uses over days 1-5, versus 10.0 in the low-dose cohort) and the highest patient-reported effectiveness, while maintaining the same favorable safety profile (no dry socket and no adverse events) as the lower doses. Taken together, these descriptive findings support the selection of the 2.0 mg/mL dose for evaluation in the future definitive trial.

Our approach aligns with emerging evidence that supports local drug delivery for post-extraction pain management. Submucosal diclofenac administration prior to third molar surgery provides effective local analgesia, with time until rescue medication ranging from 7.8 to 16 hours depending on the dose [13]. Our previous phase II study demonstrated that ibuprofen gargle significantly reduced VAS pain scores when applied to the extraction socket [15]. These findings collectively suggest that the oral extraction socket represents a viable site for local analgesic delivery.

In contrast to these earlier approaches, which employed non-selective NSAIDs delivered submucosally or as a gargle, the present study is, to our knowledge, the first to deliver a selective COX-2 inhibitor as a sustained-release paste applied directly to the socket. The dose-dependent reduction in rescue analgesic consumption observed here is consistent in direction with systemic celecoxib dental-pain studies, and the absence of impaired healing is in line with a comparative study of celecoxib, diclofenac, and ibuprofen that reported no significant differences in healing outcomes among these agents.

The choice of celecoxib as our active ingredient is supported by substantial evidence in dental pain models. An RCT demonstrates that 400 mg celecoxib provides comparable onset and magnitude of pain relief to that of 400 mg ibuprofen in postsurgical dental pain, with significantly longer duration of effect [19]. Multi-dose studies confirm that 200 mg celecoxib yields significant pain reduction and delayed time to rescue medication compared with placebo [20].

Research on orthodontic tooth movement shows that celecoxib can decrease osteoclast formation through COX-2 enzyme inhibition [21], which raises theoretical concerns about delayed socket healing. However, our findings suggest that local application at the doses tested does not impair normal healing processes, as no cases of dry socket occurred and the incidence of dry socket in the general population ranges from 0.5% to 68% depending on various risk factors [22,23]. A recent comparative study of celecoxib, diclofenac, and ibuprofen for post-extraction pain reports no significant differences in healing outcomes among these agents [24].

A systematic review and meta-analysis of analgesic combinations for postoperative pain after third molar surgery found that combinations of NSAIDs with caffeine or acetaminophen enhance efficacy with minimal AEs [25]. The American Dental Association's clinical practice guidelines recommend NSAIDs alone or in combination with acetaminophen as first-line therapy for acute dental pain management [26]. Our local delivery approach may complement these systemic strategies by providing additional analgesia at the surgical site.

Our dose range (0.5-2.0 mg/mL) was designed to achieve local therapeutic concentrations with minimal systemic exposure. Each syringe contained less than 1/100 of the maximum approved daily oral dose of celecoxib, which was substantially below systemic therapeutic levels. This conservative approach prioritized safety in this first-in-human application of a locally applied COX-2 inhibitor paste [27,28].

The integration of local COX-2 inhibitor therapy into post-extraction care could address several unmet clinical needs. A multicenter observational study shows that nimesulide is the most commonly prescribed NSAID (68%) for post-extraction pain, with notable efficacy when administered before pain onset [29]. A Cochrane review of local interventions for alveolar osteitis management identifies a significant evidence gap for preventive and therapeutic socket-applied therapies [30].

Strengths and limitations

This study had four main strengths: (i) a prospectively specified pilot design focused on healing safety with dry socket as the primary endpoint, which addressed the key theoretical concern with local anti-inflammatory therapy; (ii) dose-level exploration with consistent descriptive trends across efficacy endpoints; (iii) a clear dose-response pattern in rescue analgesic use and patient-reported effectiveness, supporting biological plausibility; and (iv) a pre-specified feasibility assessment that demonstrated the study design is practical and acceptable to patients.

However, there were six main study limitations: (i) the single-arm, open-label design without blinding or a control group limited causal inference; (ii) the small sample size (n = 18) yielded wide confidence intervals given that the incidence of dry socket can range from 0.5% to 68% depending on various risk factors; (iii) being a single-center study may limit the generalizability of findings; (iv) the five-day follow-up for efficacy measures may not capture the full duration of post-extraction pain; (v) plasma celecoxib levels were not measured, so systemic absorption remains uncharacterized; and (vi) postoperative swelling (edema), which is among the most common complications after third molar extraction, was not among the pre-specified outcome measures and was therefore not assessed in this study.

Implications for a future definitive trial

This pilot safety profile supports progression to a randomized, double-blind controlled trial. Based on our findings and the literature, we suggest five recommendations: (1) primary endpoints - co-primary endpoints of VAS area under the curve (days 1-3) and total rescue consumption consistent with established dental pain trial methodology; (2) dose selection - the 2.0 mg/mL dose for further study, based on the descriptive efficacy trends, while maintaining the favorable safety profile; (3) comparator arms - placebo paste and, optionally, an active comparator (e.g., oral celecoxib 200 mg or NSAID combination); (4) sample size - a formal power calculation based on the VAS and rescue analgesic data generated in this pilot study; (5) pharmacokinetic sub-study - to characterize local versus systemic celecoxib exposure and establish the pharmacokinetic rationale for the local delivery approach. In addition, the outcome set for the definitive trial should incorporate postoperative swelling (edema) and trismus as secondary endpoints, so that the principal complications of third molar extraction are comprehensively evaluated.

## Conclusions

The celecoxib-containing paste applied to third molar extraction sockets demonstrated favorable safety and tolerability, with no dry socket or treatment-emergent adverse events. The study design, including recruitment, retention, dose-escalation procedures, and outcome measures, proved feasible and acceptable. Exploratory analgesic outcomes show encouraging dose-dependent trends, which, taken together with the demonstrated feasibility, justify progression to a larger, rigorously controlled randomized trial to determine efficacy and establish the optimal dosing regimen for this novel local drug delivery approach.
